# Drug trapping in hERG K^+^ channels: (not) a matter of drug size?†
†The authors declare no competing interests.


**DOI:** 10.1039/c5md00443h

**Published:** 2015-12-22

**Authors:** Tobias Linder, Harald Bernsteiner, Priyanka Saxena, Florian Bauer, Thomas Erker, Eugen Timin, Steffen Hering, Anna Stary-Weinzinger

**Affiliations:** a Department of Pharmacology and Toxicology , University of Vienna , Austria . Email: anna.stary@univie.ac.at; b Department of Pharmaceutical Chemistry , University of Vienna , Austria

## Abstract

Inhibition of hERG K^+^ channels by structurally diverse drugs prolongs the ventricular action potential and increases the risk of torsade de pointes arrhythmias and sudden cardiac death. The capture of drugs behind closed channel gates, so-called drug trapping, is suggested to harbor an increased pro-arrhythmic risk. In this study, the trapping mechanisms of a trapped hERG blocker propafenone and a bulky derivative (MW: 647.24 g mol^–1^) were studied by making use of electrophysiological measurements in combination with molecular dynamics simulations. Our study suggests that the hERG cavity is able to accommodate very bulky compounds without disturbing gate closure.

## Abstract 2

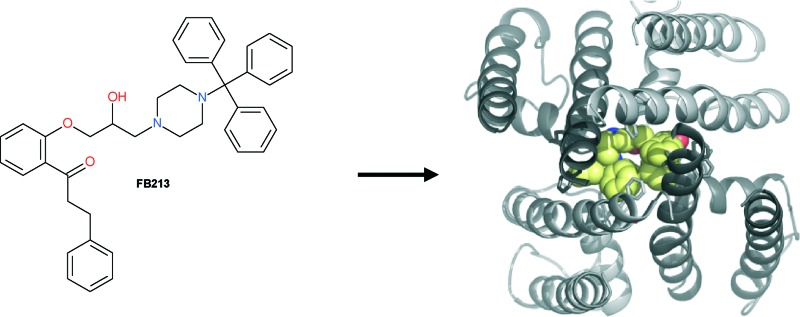
The hERG cavity can trap very bulky compounds, without perturbing normal gate closure.

## Introduction

hERG K^+^ channels (Kv11.1) are critical for the repolarization of the cardiac ventricular action potential and thus are essential for the regulation of a normal electrical heart rhythm.^1^ Loss of channel function due to inherited mutations^2^ or more commonly due to unwanted binding of small molecules^3^ can lead to long QT intervals. In the worst case scenario, this channel malfunction can cause deadly arrhythmia.^4^ These ‘off-target’ effects led to intense efforts devoted towards understanding how drug molecules physically bind to and block the pore of a hERG channel to reduce K^+^ ion flux.^5^

It was proposed that many drugs that block hERG can become trapped within the central cavity when the activation gate closes due to membrane repolarization.^6–10^ This phenomenon, referred to as ‘drug-trapping’, can explain why certain drugs cannot be washed off when channels are held in a closed state.^11^ Strong evidence for drug trapping came from studies with a mutant D540K, which can reopen at hyperpolarized membrane potentials, enabling almost complete recovery of otherwise trapped compounds.^6^

Trapping is not unique to hERG K^+^ channels and was first described for quaternary ammonium (QA) blockers by Armstrong in 1971.^12^ We have previously provided insights into the structural mechanisms of trapping of a medium size (MW: 242.46 g/mol) QA blocker. Our atomistic molecular dynamics simulations provided insights into the dynamics of the trapping process for tetrabutylammonium (TBA) in hERG K^+^ channels. Our simulations proposed that trapping can influence the dynamics of the high affinity binding determinant F656. In particular, our simulations suggested that F656 presents a physical barrier for the drug dissociation of TBA. Further, our simulations revealed that drug trapping of this compound does not influence the closure mechanism *per se*, nor does it change the structure of the gate.^13^

It was previously suggested for other K^+^ channels that larger compounds might disrupt closure of the activation gate, without really becoming trapped within the central cavity. This mechanism was termed ‘foot in the door’.^12^ This phenomenon was first described by Armstrong in 1971, where it was shown that trapping correlated with the size of QA blockers. While smaller molecules fitted into the cavity, larger QA compounds were unable to be trapped and had to dissociate before deactivation, presumably due to the limited cavity size.^12^

**Fig. 1:**
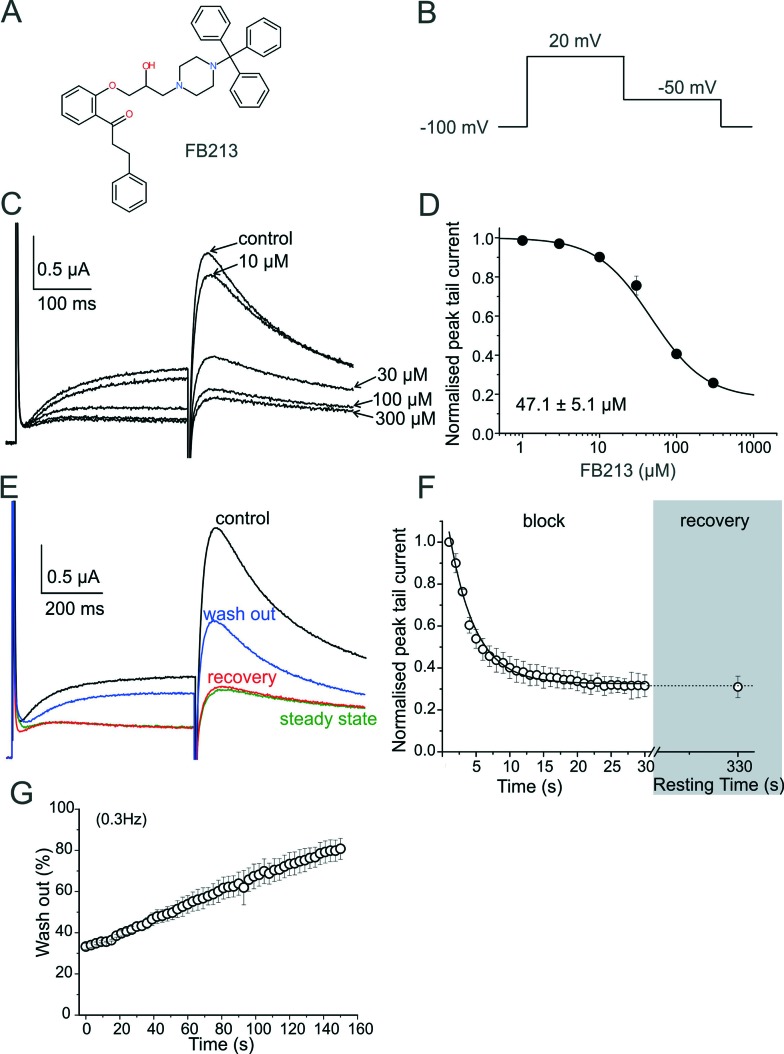
WT hERG channels inhibition by Fb213. (A) Chemical structure of FB213; (B) voltage pulse protocol shown; (C) superimposed current traces recorded in the absence (control) and after attaining a steady-state block with increasing concentrations of FB213 at 0.3 Hz; (D) the concentration–response relationship for the block of the hERG tail current by FB213; (E) superimposed current traces of the first (control, black) and last (‘steady-state block’, green) pulses during a conditioning train of 1 Hz after application of 150 μM FB213 Recovery current from the FB213 block in the continued presence of drug at rest, resulting from a single test pulse after 330 s resting time is depicted as red, washout of the FB213 block is shown in blue; (F) mean normalized peak tail current amplitudes in the presence of 150 μM FB213 is plotted against time. The section ‘block’ shows the development of inhibition during a 1 Hz pulse train. The grey highlighted section ‘recovery’ maps the amount of recovery after a 330 s resting time; (G) repetitive stimulation accelerates wash-out of FB213. The hERG channels were inhibited by a 1 Hz pulse train, as described in Fig. 1F. After reaching steady state of inhibition, the drug was washed out. During the wash-out process, pulses were applied at 0.3 Hz frequency. Peak tail currents were normalized to control the currents (amplitude before drug application) and plotted against time.

The hERG K^+^ channel cavity is rather unique, since it is able to bind many structurally diverse chemicals of various sizes with high affinity.^5^ In order to investigate if larger compounds influence or prevent proper channel closure in hERG, we made use of a well-studied class Ic antiarrhythmic drug, propafenone,^14^ which is known to be trapped in the hERG channel,^8^ and synthesized a novel bulky derivative (Fig. 1A). By combining two-electrode voltage clamp analysis and molecular dynamics simulations, we provide detailed insights into drug trapping in relation to compound size in hERG channels.

## Methods

### Synthesis of 1-[2-(2-hydroxy-3-(4-tritylpiperazin-1-yl)-propoxy)phenyl]-3-phenylpropan-1-one (FB213)


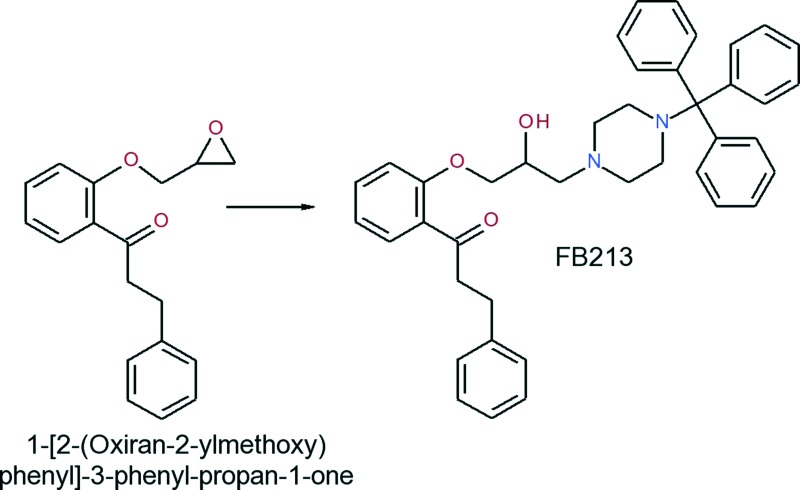
All chemicals obtained from commercial suppliers were used as-received and were of analytical grade. Melting points were determined on a Kofler hot stage apparatus and were uncorrected. ^1^H- and ^13^C-NMR spectra were recorded on a Bruker Avance DPx200 (200 and 50 MHz).

A mixture of 1-[2-(oxiran-2-ylmethoxy)phenyl]-3-phenyl-propan-1-one (0.576 g, 2.04 mmol) and 1-tritylpiperazine (0.688 g, 2.09 mmol) in 2-propanol was heated to reflux for 6 hours. Upon completion of the reaction, the solvent was evaporated and the residue was recrystallized from ethyl acetate to obtain the title compound as a white solid (yield: 0.854 g, 68.5%).

The analysis of this material gave the following results: Mp 162–165 °C; ^1^H-NMR (CDCl_3_): 7.78–7.65 (m, 1H), 7.58–7.10 (m, 21H), 7.05–6.85 (m, 2H), 4.18–3.81 (m, 3H), 3.58–2.20 (m, 10H), 1.60 (s-br, 2H); ^13^C-NMR (CDCl_3_): 201.4, 158.0, 141.8, 133.6, 130.6, 129.5, 128.5, 128.3, 127.7, 126.2, 126.0, 121.1, 112.7, 77.0, 70.9, 65.2, 61.0, 47.9, 45.9, 30.4. MS *m*/*z* 411 [0.1%], 125 [13%], 99 [100%]. Anal. calcd for C_41_H_42_N_2_O_3_: C, 80.62; H, 6.93; N, 4.59. Found: C, 80.41; H, 6.83; N, 4.55.

### Electrophysiology

All experiments involving animals were approved by the Austrian Animal Experimentation Ethics Board in accordance with the European convention for protection of vertebrate animals used for experimental and other scientific purposes ETS no. 123. cDNAs of hERG (accession number NP000229) was kindly provided by Prof. Sanguinetti (University of Utah, UT, USA). Synthesis of capped runoff complementary ribonucleic acid (cRNA) transcripts from linearized cDNA (cDNA) templates and injection of cRNA were performed as described in detail by Sanguinetti *et al.* ^2^ Oocytes from a South African clawed frog, *Xenopus laevis* (NASCO, Fort Atkinson, WI, USA), were prepared as follows: after a 15-min exposure of a female *Xenopus laevis* to an anesthetic (0.2% solution of MS-222, the methanesulfonate salt of 3-aminobenzoic acid ethyl ester; Sigma), parts of the ovary tissue were surgically removed. Defolliculation was achieved by enzymatic treatment with 2 mg mL^–1^ collagenase type 1A (Sigma) and mechanical removal of the follicular layer using forceps. Stage V–VI oocytes were selected and injected with the WT and mutant hERG-encoding cRNA. Injected oocytes were stored at 18 °C in ND96 bath solution (96 mM sodium chloride, 2 mM potassium chloride, 1 mM magnesium chloride, 5 mM HEPES, 1.8 mM CaCl_2_; pH 7.5, titrated with NaOH) containing 1% penicillin-streptomycin solution. All chemicals used were purchased from Sigma-Aldrich Chemie GmbH, Taufkirchen, Germany.

Currents through the hERG channels were studied 1 to 4 days after the microinjection of cRNA using the two-microelectrode voltage clamp technique. ND96 was used as the extracellular recording solution. Voltage-recording and current-injecting microelectrodes were filled with 3 M KCl and had resistances between 0.3 and 2 MΩ. Endogenous currents (estimated in oocytes injected with DEPC water) did not exceed 0.15 μA. Currents >5 μA were discarded to minimize voltage clamp errors. Ionic currents were recorded with a Turbo Tec 03X amplifier (npi electronic, GmbH, Tamm, Germany) and digitized with a Digidata 1322A (Axon Instruments Inc., Union City, CA, USA). The pClamp software package version 9.2 (Axon Instruments Inc.) was used for data acquisition. Microcal Origin 7.0 was employed for analysis and curve fitting.

A precondition for all the measurements was the achievement of stable peak current amplitudes over periods of 10 min after an initial run-up period. A frequency of 0.3 Hz was used for all the voltage clamp experiments. Drugs were applied by means of a perfusion system enabling solution exchange within 100 ms.^26^ Control measurements for 30–35 minutes after an initial ‘run up’ phase were performed and no significant changes in current amplitude were observed. The flow rate was ≈8 μl s^–1^, preventing run down during the experiments (data not shown). The oocytes were kept for 5 min at a holding potential of –100 mV to equilibrate drug diffusion. The tail current was measured at –50 mV after a step to +20 mV. A use-dependent hERG channel block was estimated as the peak tail current inhibition. Data are presented as means ± s.e. from at least four oocytes from ≥2 batches. The studied compound (FB213) was dissolved in the ND96 extracellular recording solution to prepare a 10 μM stock on the day of the experiments. The drug stock solution was further diluted to the required concentration. All experiments involving animals were approved by the Austrian Animal Experimentation Ethics Board in accordance with the European convention for protection of vertebrate animals used for experimental and other scientific purposes ETS no. 123.

### Molecular docking

The hERG homology model termed “model 6” from our recently published analysis of structural hERG models was used for docking.^18^ The modeling procedures and validation are described in detail in our previous paper. Briefly, Modeller 7v7 (ref. 29) was used to generate a 3D model of the open conformation of hERG1, based on the crystal structure of KvAP^30^ and the refined model thereof.^31^

Docking was performed using the program Gold 4.0.1 and the implemented Gold scoring function. The binding site was defined by selecting Y652 and F656 of all four subunits as the center and including all residues within a radius of 10 Å of these two residues. The Gold rotamer library was used to account for side chain flexibility.^15^ For both drugs, the central nitrogen was protonated and used in its charged form. The 20 best-ranked poses of each drug docking run were visually inspected and the most frequent binding mode was used as the starting conformation for ED simulations. General amber force field parameters^16^ for the drugs were generated by making use of Gaussian 09 (ref 27) and antechamber.^28^

### Essential dynamics simulations (ED)

The ED technique was described previously.^17^ Briefly, an eigenvector representing the transition between open and closed hERG channel states was obtained from a principal component analysis by comparing the backbone atoms of both states. The fixed increment linear expansion method was used and set to –1.69 × 10^–6^ nm per simulation step (2 fs). Five closing ED simulations, each lasting 20 ns, were performed in the presence of propafenone and FB213. Data for apo simulations were taken from our previous publication.^13^

## Results and discussion

### FB213 inhibits hERG currents and is trapped in the cavity

hERG channels were expressed in *Xenopus laevis* oocytes and K^+^ current was measured using the standard two-electrode voltage clamp technique. A two-step pulse protocol was applied (Fig. 1B): 300 ms depolarization to +20 mV (prepulse) induces slow activation and fast inactivation of the channels, and potassium current during prepulse is small. Upon repolarization to –50 mV, the channels underwent rapid recovery from inactivation, inducing large “tail” currents, and were slowly deactivated (closure of the channel gate). Peak tail current amplitudes were used as a measure of the fraction of channels free from drug inhibition. First, we determined the sensitivity of FB213 to the WT hERG channels. A concentration inhibition relationship was estimated by plotting the steady state values of current inhibition normalized to control (in a drug-free solution) *versus* cumulatively applied FB213 concentrations (Fig. 1C and D). The concentration of FB213 required to block 50% (IC_50_) of the hERG current was 47.1 ± 5.1 μM. The oocyte was exposed to the drug and after 5 min of equilibration, a train of pulses (with a frequency of 1 Hz) was applied, inducing “use-dependent” inhibition of the hERG channels (Fig. 1F, block): peak tail currents were gradually decreased and finally reached a steady state.

A state-dependent block was measured in the absence of FB213 (control, Fig. 1E) and after a pre-incubation period of 330 s with 150 μM FB213 (3 × IC_50_) while holding the channels at –100 mV. Subsequently, 1 Hz pulse trains were applied until a steady state block was reached. A channel block was developed in a ‘use-dependent’ manner. Prepulse and tail currents were inhibited during the 1 Hz pulse train. The steady state block was achieved within 15 s (Fig. 1E green trace and F). The development of the block during the channel activation at +20 mV suggests that FB213 blocks the hERG channels in an open channel conformation. 150 μM FB213 blocked the hERG channels by 69 ± 5% (Fig. 1F).

Further, we probed if FB213 is trapped inside the hERG cavity. The criteria of drug trapping in the hERG channel cavity are: i) lack of recovery from the block at rest and ii) slow recovery and acceleration of recovery during washout of the drug.^9^ Recovery from the hERG channel block by FB213 was determined by applying a test pulse after a resting period of 300 s at a holding potential of –100 mV, where the channels are in a closed resting state. The first current amplitudes after this rest period recovered from the block are less than 1%, indicating that FB213 is trapped in the closed channel conformation (Fig. 1E red trace and F). Previously, it has been demonstrated that hERG channels inhibited by “non-trapped” drugs recover during ≈330 s even in the presence of drugs.^9,10^ Subsequent frequent opening of the channel at 0.3 Hz during wash out induced substantial recovery from the FB213 block to 47.5 ± 3.7% (Fig. 1E blue trace and G), suggesting that trapped FB213 can leave the channel during activation when the channels are in an open conformation.

**Fig. 2:**
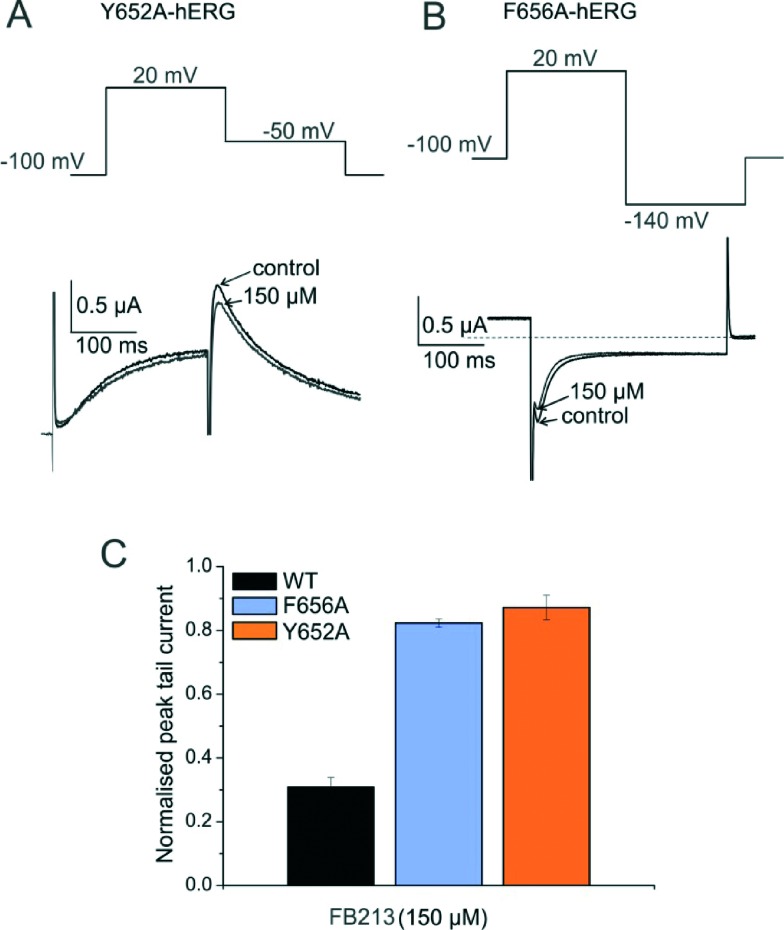
FB213 interacts with binding sites Y652 and F656. (A, B) Representative current traces and corresponding voltage protocols for current measurements of mutants Y652A and F656A in the absence (control) and presence of FB213, respectively. The tail currents of F656A were recorded at –140 mV. (C) Normalized peak tail currents of WT, Y652A, and F656A channels after the steady state block by 150 μM FB213 (*n* = 4, error bars, ± SEM).

To test if FB213 binds to the central cavity, as shown previously for other propafenone derivatives,^10^ we performed alanine mutation studies on Y652 and F656, which have been shown to play a key role for the binding of different chemical entities.^6^ The WT channel voltage protocol was utilized for Y652A, while tail currents were measured at –140 mV for F656A, as reported by Witchel *et al.* ^8^ Y652A and F656A significantly reduced channel inhibition to 13.2 ± 4.4% and 18.3 ± 1.2%, respectively (Fig. 2A–C), suggesting that FB213 not only can access the binding site inside the cavity, but further interacts with Y652 and F656, as shown for many well-known hERG blockers.

Taken together, these results suggest that FB213 can bind to the well-established receptor site located deeply in the channel pore and does not dissociate from the channel at rest. Washing out provided negligible recovery from the block which is prominently enhanced by frequent stimulation.

### Structural investigation of FB213 block in hERG

**Fig. 3:**
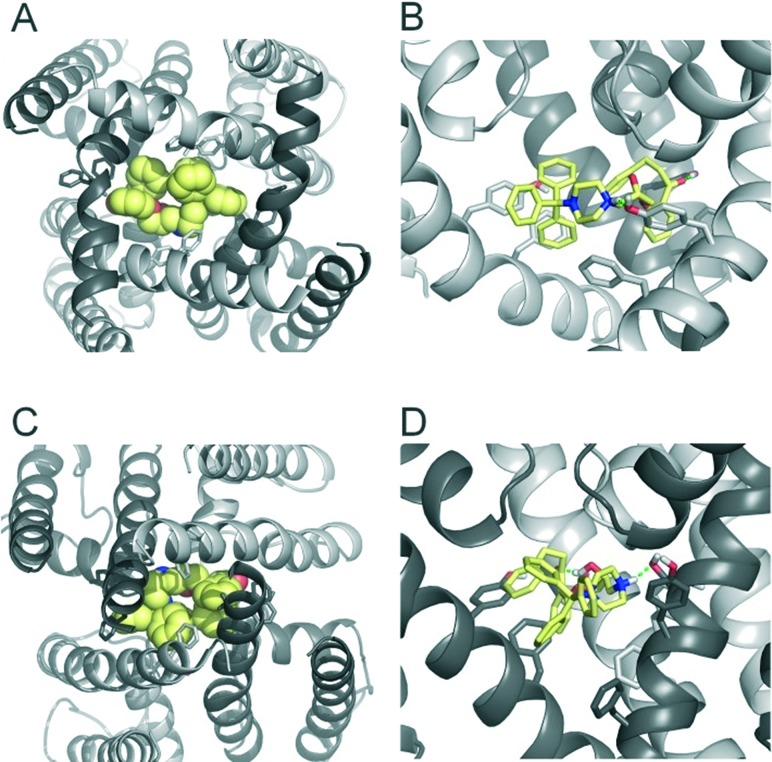
Modelling of FB213 trapping. (A) Bottom view of FB213 (yellow spheres) docked into the open state hERG model. (B) Side view of FB213 interactions with the aromatic side chains of Y652 and F656. Hydrogen bonds are depicted as green dotted lines. (C) Bottom view of the trapped FB213 after 20 ns ED simulations. (D) Side view of the compound in the closed state.

To investigate the binding mode of FB213, we docked the compound into our previously published open state hERG homology model.^18^ Hydrogen bonds between two adjacent hydroxyl groups of Y652 and the drug were observed. The protonated nitrogen of FB213 is located beneath the selectivity filter, stabilized by helix dipole charges and a hydrogen bond to Y652. The triphenyl moiety of the compound forms hydrophobic and aromatic interactions with two adjacent Y652 side chains and one F656 residue. As illustrated in Fig. 3B, multiple hydrophobic and aromatic interactions between the compound and the side chains of Y652 and F656 were observed. This is in agreement with our experimental observations.

**Fig. 4:**
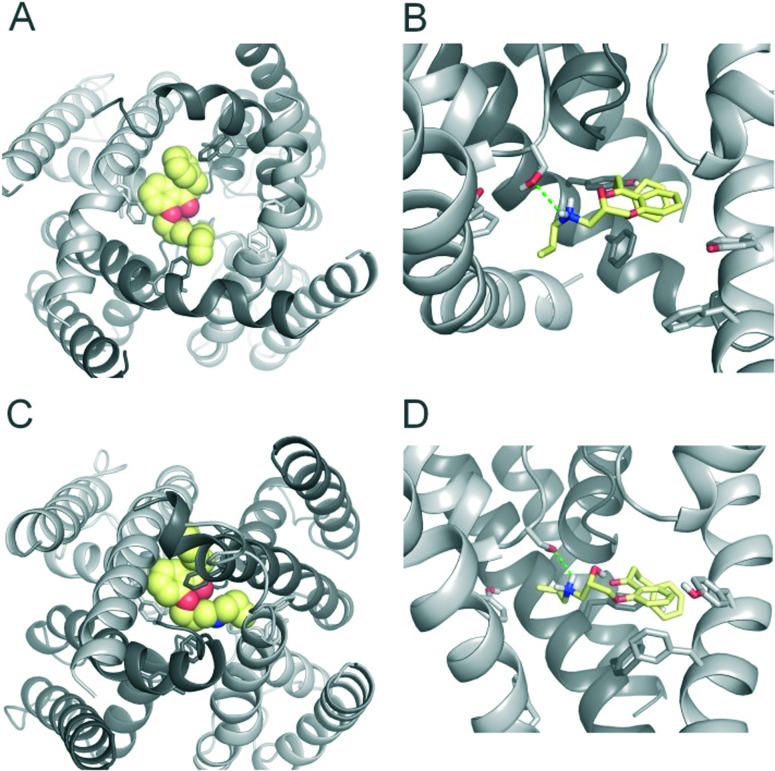
Modelling of propafenone trapping. (A) Bottom view of propafenone shown in yellow spheres docked into the open state hERG model. (B) Side view of propafenone interactions with the side chains of Y652, F656 and S624. Hydrogen bonds are depicted as green dotted lines. (C) Bottom view of the trapped propafenone molecule after 20 ns ED simulations. (D) Side view of the compound in the closed state.

For comparison reasons, we included the well-studied smaller propafenone (MW: 341.44 g mol^–1^) compound in our ED investigations as well. As a starting point, the molecule was docked into the open state, similar to FB213. The interactions are illustrated in Fig. 4B. In agreement with the experiment and previous docking studies,^8,19^ aromatic and hydrophobic interactions between propafenone and F656 and Y652 were observed. Additionally, a hydrogen bond between the basic nitrogen and S624 was seen.

### Trapping simulations with propafenone and FB213

To investigate if propafenone and FB213 can indeed become physically trapped behind the activation gate, we performed five independent essential dynamics (ED) gating simulations with both drugs. The ED method was previously successfully applied by our group to investigate activation/deactivation gating in KcsA^17^ and to monitor drug trapping of TBA in KcsA and hERG.^13^ In the first step, we compared the backbone atoms of the open and closed state hERG homology models by principal component analysis. The resulting eigenvector was then used to enforce channel closure, while leaving all other degrees of freedom essentially unbiased. The simulations enabled us to monitor drug trapping in atomistic detail on the nanosecond timescale. Closure of the activation gate was monitored by calculating the RMSD (root mean square deviation) of the protein compared to the closed state homology model. As shown in Table 1, the RMSD value decreased steadily, reaching minima between 2.33 and 3.51 Å. The somewhat higher RMSD values in two closing simulations (3.51 Å and 3.13 Å) resulted from unwinding of the helix termini. However, this was not influenced by the bound drug molecule, since the binding site was higher up in the cavity (see Fig. 3A–D). Successful trapping was defined by a decrease in the RMSD and visual inspection of the gating region formed by S6 segments. As listed in Table 1, all 10 closing runs with the drugs were successful. Our ED simulations repeatedly show that the activation gate can close normally with FB213, as illustrated in Fig. 3C and D.

**Table 1:** Analysis of closing simulations. Minimal root mean square deviation (MinRMSD) and time to reach the closed state are listed

Closing simulations					
FB213		Apo		Propafenone	
MinRMSD (Å)	Time (ns)	MinRMSD (Å)	Time (ns)	MinRMSD (Å)	Time (ns)
3.13	16.2	2.90	15.2	2.81	19.7
2.33	18.4	2.92	14.5	2.79	18.9
3.07	9.7	3.41	10.7	3.51	8.3
2.74	13.2	3.15	14.2	2.38	18.0
2.62	14.9	2.62	18.7	2.79	16.7

**Fig. 5:**
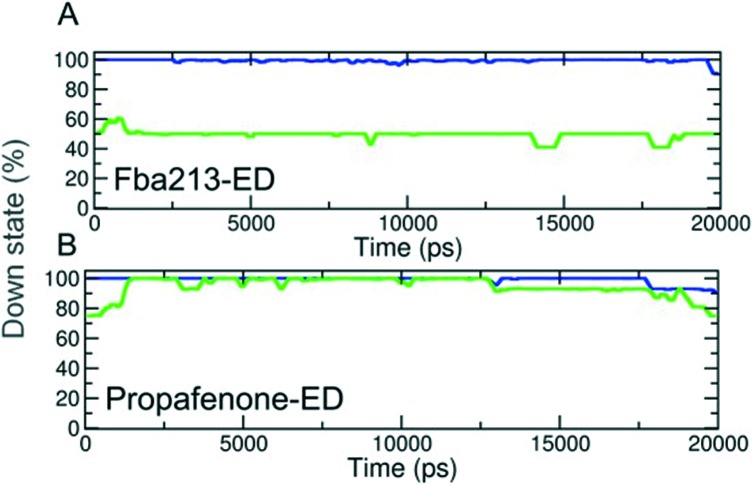
Analysis of rotameric states of F656. (A–B) Down states are defined by *χ*1 angles ≤123°. The percentage of the down state was calculated for each time step and plotted over time averaged over 5 trapping runs/drug.

Since we have previously found that closure with a bound drug molecule can influence the dynamics of the F656 side chain,^13^ we monitored the rotameric states of this residue during closure simulations. As shown in Fig. 5, propafenone as well as FB213 influence the dynamics of the side chain. In particular, the bound propafenone stabilizes the down state of F656, which is defined as *χ*1 angles ≤123°. Only at the end of the simulation, the side chain of 1 subunit is found in the up-state (*χ*1 ≤123°). These effects are similar as observed for TBA trapping in our previous study.^13^ The situation is different for FB213. Due to the large size of this molecule, the behaviour of the F656 side chain is influenced. At the start, only two F656 side chains are able to adopt a down-conformation, while the other two side chains have to adopt an ‘up-ward’ conformation. In all five closing simulations, this distribution of F656 rotameric states does not change during closure. (Fig. 5).

During channel closure, the interactions between FB213 and the protein increase, compared to the observed open state interactions (Table 2). Due to the rotation of the S6 helix during closure, the side chain of S649 is reoriented, enabling hydrogen bonds with the protonated nitrogen, additionally to the OH–group of Y652 as shown in Fig. 3B and D. The location of the tertiary amine is only slightly different from the open state. This is in contrast to a previous docking study which suggested that the basic nitrogen might move upward during channel closure.^19^

**Table 2:** Analysis of the buried solvent-accessible surface area of the ligand. Calculations were performed with Surface Racer 5.0 (ref. 32)

Buried solvent accessible surface areas (= SASA, Ångström^2^)			
	Total buried area	Polar	Non-polar
FB213			
Run 1	450.17	130.3	319.86
Run 2	414.16	104.08	310.09
Run 3	443.52	128.47	315.05
Run 4	408.39	117.35	291.06
Run 5	456.21	126.36	329.84
Average buried SASA	434.49	121.31	313.18
			
Propafenone			
Run 1	212.9	80.04	132.87
Run 2	1.12	0.35	0.77
Run 3	203.11	30.91	172.21
Run 4	382.15	112.82	269.33
Run 5	258.01	94.4	163.59
Average buried SASA	211.45	63.70	147.75

During channel closure with propafenone, no major changes in the interactions compared to the open state were observed (Fig. 4B and D). Again, the basic nitrogen remained centrally located below the selectivity filter, stabilized by helix dipole charges. As expected, due to size differences, the buried solvent accessible surface areas of FB213 compared to propafenone is considerably higher, as shown in Table 2. Interestingly, in ED run 2, propafenone did not displace any solvent from the surface areas of the protein. In this run, the ligand is forming a U-shaped conformation. This is in agreement with a recent study by Schmidtke *et al.*,^20^ suggesting that drug interactions can become more favourable when the cavity size is decreased. During channel closure, we often observe a 2-fold symmetry, irrespective of the drug bound. This is also in line with closure simulations performed recently on hERG homology models without drug molecules.^20^

It is remarkable that a molecule the size of FB213 seems to be accommodated in the hERG cavity without any difficulties. This suggests that the cavity of hERG is quite different from other potassium channels with smaller cavities, such as Shaker.^21^ This underlines the uniqueness of the hERG cavity in terms of size and possibly plasticity, which has recently been recognized to play a major role in drug block as well.^15,20,22,23^ It is conceivable that the hERG cavity might be able to accommodate even larger/bulkier molecules when considering hydrophobic side pockets, which have been recently suggested to be accessible for drug interactions.^20,22,24^ Interestingly, such a possibility has recently also been shown for a QA blocker in the bacterial K^+^ channel KcsA.^25^

## Conclusions

Our data suggests that pore blockers of different bulkiness may serve as tools to probe the size of the HERG cavity.

We found that even large blockers do not hinder normal gate closure. This indicates that the cavity of the hERG pore is remarkably large, enabling trapping of compounds with very high molecular weight (FB213: 647.24 g mol^–1^). Further, we propose that for a propafenone-like scaffold, size does not play a major role during drug trapping in hERG K^+^ channels. Further studies will reveal if this holds true for structurally unrelated trapped drugs in hERG.
